# Editorial on the Themed Issue in Honor of Carlos Gutiérrez Merino: Forty Years of Research Excellence in the Field of Membrane Proteins and Bioenergetics

**DOI:** 10.3390/molecules30081710

**Published:** 2025-04-11

**Authors:** Alejandro K. Samhan-Arias, Carmen López-Sánchez, Manuel Aureliano

**Affiliations:** 1 Departamento de Bioquímica, Universidad Autónoma de Madrid (UAM), C/Arturo Duperier 4, 28029 Madrid, Spain; 2 Instituto de Investigaciones Biomédicas Sols-Morreale, Consejo Superior de Investigaciones Científicas-Universidad Autónoma de Madrid (CSIC-UAM), 28029 Madrid, Spain; 3 Department of Human Anatomy and Embryology, Faculty of Medicine and Health Sciences, Institute of Molecular Pathology Biomarkers, University of Extremadura, 06006 Badajoz, Spain; clopez@unex.es; 4 Faculdade de Ciências e Tecnologia (FCT), Universidade do Algarve, 8005-139 Faro, Portugal; 5 Centro de Ciências do Mar (CCMar), Universidade do Algarve, 8005-139 Faro, Portugal

Prof. Carlos Gutiérrez-Merino (Figure 1) has led over 30 research projects funded by national and international agencies and, under his guidance, numerous researchers have developed their doctoral theses, contributing to the growth of biomedical research in Extremadura. His career has been dedicated to the investigation of several topics, including calcium homeostasis in muscle and neuronal cells, oxidative stress, and cellular bioenergetics with a focus on proteins and biomolecules such as P-type ATPases and muscle proteins [1,2,3,4]; the neuronal plasma membrane L-type calcium channels [5,6], and its role in neurodegeneration, including Alzheimer’s disease [7,8,9,10]; microsomal reductases function in oxidative stress [11,12,13,14]; the role of natural antioxidants [15,16,17,18]; and the toxicology of vanadium [19,20].

The impact of his scientific work is reflected in more than 130 impact publications, and his participation in books and international conferences. His research has been widely cited, and has inspired new lines of study in biochemistry and neuroscience. His work has been instrumental in establishing the University of Extremadura as a reference center for biomedical research, and in positioning Extremadura on the national and international scientific map. Prof. Gutiérrez-Merino remains active in research, and maintains collaborations on projects with colleagues and friends that we want to dedicate this editorial letter to: Prof. Manuel Aureliano (University of Algarve, Portugal), Prof. Carmen López-Sánchez (University of Extremadura, Spain), and Prof. Alejandro K. Samhan-Arias (Autonomous University of Madrid, Spain).

In this Special Issue, “Themed Issue in Honor of Prof. Carlos Gutiérrez-Merino: Forty Years of Research Excellence in the Field of Membrane Proteins and Bioenergetics”, a wide range of topics are addressed, including research directly related to his work, studies derived from his research conducted by his disciples, and contributions from collaborators and friends who wished to dedicate their work to him in this Special Issue. These areas include: Fluorescence Resonance Energy Transfer (FRET) to high-resolution cell imaging [21]; molecular mechanisms of trabectedin and lurbinectedin, alkaloid compounds originally isolated from *Ecteinascidia turbinata*, to induce cell death in tumoral cells [22]; selenocysteine-containing proteins [23]; extracts or isolated compounds for therapy against neurodegenerative diseases [24]; proteins that play major roles in calcium signaling [25]; plasma membrane raft-like domains operate as hubs for toxicants’ cellular actions [26]; and membrane lipid derivative arachidonic acid and its metabolites in the development of pancreatitis and diabetes [27].

Moreover, six regular papers from different research areas were published at this Special Issue, highlighting: (1) the discovery of new routes for polycyclic aromatic hydrocarbons (PAHs’) toxicity to mitochondria, highlight the importance of mitochondrial membranes and cytochrome *c* in bioenergetics and environmental detoxification [28]; (2) the description of the His_6_-tag as a molecular target for neurotoxic Aβ peptides, suggesting its use to direct the action of these peptides toward selected cellular targets [29]; (3) the description of the role of store-operated calcium entry inhibition and the plasma membrane calcium pump in the maintenance of resting cytosolic calcium levels, leading to an increased production of amyloid precursor protein (APP) and Aβ peptides in A1-like astrocytes [30]; (4) a study of the vesicle mechanical behavior upon its exposure to 3-hydroxybutyric acid, using an atomic force microscope (AFM) [31]; (5) a description of the interaction of Lysyl oxidase-like 2 with numerous RNA-binding proteins, emphasizing the complexity of protein interactions in cellular signaling and cancer biology [32]; and (6) guidelines for resazurin reduction-based assays, opening new avenues to integrate chemistry, biology, toxicology, and pharmaceutical science into standardized and comparable applications for the resazurin assay [33]. So far (19 March 2025), these six papers and seven reviews have garnered a total of 85 citations and 41193 views, indicating an average of 7 citations and 3168 views per publication.

At the time at which he celebrated almost 50 years of active research since he started his PhD thesis, we wanted to celebrate his academic and research achievements, but also his human and professional legacy. His example of perseverance, rigor, and passion for science has left an indelible mark on those who have had the privilege of working and following him. We want to extend our deepest recognition and gratitude to Prof. Carlos Gutiérrez-Merino. His career stands as a testament to how dedication and effort can transform research with limited resources such as Extremadura, and turn them into benchmarks of scientific excellence. As recent contributions from Prof. Carlos Gutiérrez-Merino also include the direction several doctoral theses, whose more relevant results have been published in this Special Issue and in Molecules (MDPI) [25,29,30]. The most recent discovery of several polyoxometalates (POMs) as inhibitors of SERCA/PMCA, as well as agonists of ionotropic and metabotropic receptors in neurons [34,35], reflects indeed his perseverance, rigor, and passion for science.

Putting it all together, Prof. Carlos Gutiérrez-Merino has been responsible for a new generation of scientists across Spain and Portugal for four decades. Prof. Gutiérrez-Merino trained started to go deep into the understanding of molecular interactions of biomolecules with enzymes during their PhD studies. Fluorescence Resonance Energy Transfer (FRET) was used to study lipid state transitions and proteins distribution in membranes. Prof. Gutiérrez-Merino brought his knowledge to Spain, being the mentor of many students in interdisciplinary fields of biochemistry and biophysics, shaping the future of a new generation of scientists and professors. Prof. Gutiérrez-Merino has also promoted several collaborations with Portugal, allowing many students and professors to be involved in his wonderful mentoring. Emergent topics arose in this time, such as protein targets for nitrosative and oxidative stress, cross-talk between Ca^2+^ homeostasis and redox pathways in neurological diseases, antioxidants for cell protection, and disease prevention and polyoxometalates as agonists of ionotropic and metabotropic receptors and inhibitors of SERCA and PMCA, among many others. Altogether, Prof. Carlos Gutiérrez-Merino, shaped the future of many researchers in Spain and Portugal, and made a major and unique contribution to the advance of the knowledge in several interdisciplinary fields of biochemical sciences.

## Figures and Tables

**Figure 1:**
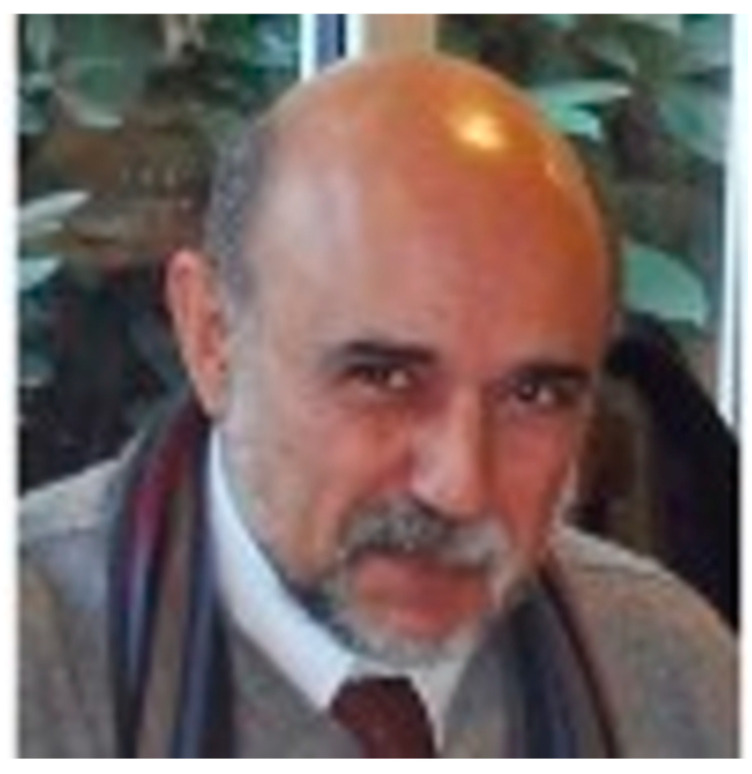
Prof. Carlos Gutiérrez-Merino (kindly provided by Carlos Gutiérrez-Merino).
